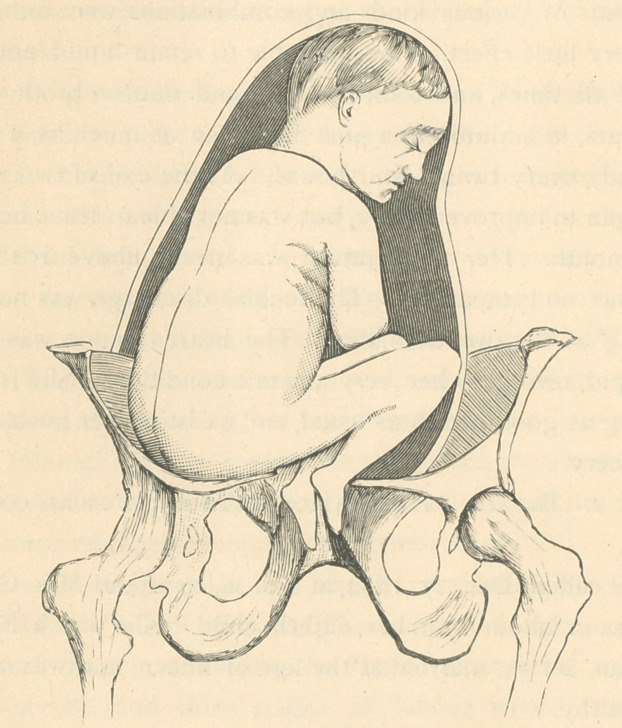# Two Interesting Cases in Obstetrics

**Published:** 1885-04

**Authors:** Charles Caldwell

**Affiliations:** 815 Forty-third Street


### ARTICLE III.

### TWO INTERESTING CASES IN OBSTETRICS.

*By* CHARLES CALDWELL, A. M., M. D.

**[Inaugural Thesis read before the Chicago Gynecological Society, Feb. 20th, 1885.]**

CASE I. ANTE-PARTUM HÆMORRHAGE, CAUSED BY ACCIDENTAL SEPARATION OF THE PLACENTA.

Mrs. B——, German, aet 45, and pregnant with her eleventh child, was awakened at 1 a. m. Sept. 19th, 1884, by an acute pain in the epigastric region, followed by a perfect gush of blood from her vagina. A neighbor midwife was called, but became frightened at the profuse hæmorrhage and advised that a physician be sent for. I reached the house at 3 A. M. The midwife said she had been in attendance an hour, and had removed several sheets and quilts which were saturated with blood.

Fearing placenta prævia, I made an examination at once. The os was dilated the size of a silver dollar, but the head, which was presenting, was high up and not yet engaged. The membranes were still intact. I passed my finger up into the uterus, and found it was not even a case of placenta prævia marginalis. The uterine contractions were weak and ineffective, but the pain in the epigastric region still acute and incessant.

She said her suffering was greater than at any previous confinement, and very different; this was more like colic pain.

Her husband is a grocer, and she had been assisting him in the store during her period of gestation, handling boxes and baskets of heavy vegetables and fruits.

I diagnosed the case one of partial or complete detachment of the placenta, brought about by lifting some large, heavy body, which she had pressed against her abdomen. For we know washerwomen often bring on miscarriage by lifting their heavy tubs.

I gave ergot immediately, and pressed on the fundus with the left hand, to force the head down into the pelvis, and thus check the hæmorrhage, while with the right I dilated the os. The uterus did not respond readily to the ergot, for the contractions did not increase in strength or frequency, and a domestic remedy was tried. A bag of hot salt, four inches wide by twelve inches long, was applied to the spine. Whether the salt deserves the credit by its stimulating effect, or the ergot was tardy in its action, I am unable to say, but within twenty minutes after the application of the salt, the uterine contractions had so increased in force, that the head had descended and become engaged, stopping the hæmorrhage.

Pains were now regular and strong, completing the second stage of labour in half an hour. The membranes were not ruptured until ten minutes before the fœtus was expelled.

The second and third stages of labour were completed together, and the maternal surface of the placenta was covered with a dark, thick blood clot, showing it had been detached some time. The child was still-born. From the history of this case, it comes under class (b) as described by Dr. Goodell, in his monograph on "CONCEALED ACCIDENTAL HÆMORRHAGE OF THE GRAVID UTERUS," where the placenta is so detached as to allow the blood to escape behind the membranes into the uterine cavity. After the uterus had expelled its contents it contracted well, and gave my patient no more trouble, which was very fortunate for her. She was safely through her parturition, but by no means out of danger, for the hæmorrhage had been so profuse that it left her exsanguinated and exhausted.

The third day after her confinement, diarrhœa set in, and for ten days she passed copious involuntary stools. Opiates and astringents of various kinds and combinations were exhibited with very little effect. She was able to retain liquid nourishment at all times, and took her milk and mutton broth every two hours, in addition to a pint and even as much as a quart of brandy every twenty-four hours. At the end of two weeks she began to improve slowly, but was not able to leave her bed for a month. Her temperature was never above 101°, and there was no tympanites. The lochial discharge was normal, stopping at the twentieth day. The heart's action was weak and rapid, owing to her very anæmic condition. She is now enjoying as good health as usual, and assisting her husband in his grocery.

CASE 2. BREECH PRESENTATION WITH AN UNUSUAL COMPLICATION.

I was called, Dec. 27, 1884, at 4 A. M., to attend Mrs. C——, who was in labour with her eighth child. She was a French Canadian, aet 31, married at the age of fifteen, and was strong and healthy.

On my arrival, she told me "she had been in labour two hours and that the waters had broken before she had her first pain."

As soon as I could warm and disinfect my hands, I made an examination. The os was dilated to the size of a small doorknob, soft and patulous.

Both feet were presenting and protruding through the os. By passing the index finger up into the uterus, above the brim, the tuberosities of the ischia, vulva and anus of the fœtus could be felt to the right of the median line. By palpation the knees could be felt above the brim on the left. The fœtus seemed to be seated on the right brim of the pelvis with her knees resting on the left, sticking her feet down through the os uteri for the accoucheur to seize hold of.

That this peculiar and unique position was an impediment to labour, no one can doubt, if the diagram is a correct one.

Whether the second stage of labour could have been completed without assistance it is impossible to say, but I think not.

The *liquor amnii* had all escaped, so I dilated the os with my hand, completing the first stage of labour as quickly as possible. By bi-manual manipulation I changed the position of the fœtus, so that it was possible to bring down the feet, in the following way:

I introduced my right hand into the vagina, and the index and second fingers into the uterus; placing the ends of the fingers against the tuberosities of the ischia, I made pressure upwards and to the right, while with the left hand over the fundus uteri, I made pressure on the occiput of the fœtus, forcing the head downwards and to the left. Holding the fœtus in its new position with the left hand, I easily brought down the feet with my right. While I was delivering the body of the fœtus, I asked one of the attendants to warm a towel to wrap around the child while I was delivering the head, expecting some delay, for it was quite large. As I was in the act of wrapping the towel around the child, the mother, with one mighty muscular effort and a scream, expelled the head, making me feel happier than any one present except the mother.

I never saw a woman who had as much expulsive power as this one.

I attended her three years ago, when her seventh child was born, and she was in labour only twenty minutes, having but four pains.

When I made the first examination, she asked me "if her baby was in a natural position," and upon being told it was a foot presentation, she said, "she had been afraid something was wrong, for she had felt the movement of the child on the left side, while with all the others it had been on the right. She left her bed the third day after her confinement (not by my advice), and has been doing all of her work since. In examining the various obstetric works at my command, I have failed to find the report of a case with this peculiar complication.

815 Forty-third Street.

## Figures and Tables

**Figure f1:**